# The relationship of income on stroke incidence in Finland and China

**DOI:** 10.1093/eurpub/ckad035

**Published:** 2023-04-22

**Authors:** Honghui Yao, Liina Junna, Yaoyue Hu, Xinping Sha, Pekka Martikainen

**Affiliations:** Department of Learning, Informatics, Management, and Ethics, Karolinska Institutet, Stockholm, Sweden; Department of Infectious Diseases, ChangdeXiangya Hospital, Central South University, Hunan, China; Population Research Unit, Faculty of Social Sciences, University of Helsinki, Helsinki, Finland; The Max Planck Institute for Demographic Research, Rostock, Germany; School of Public Health and Management, Chongqing Medical University, Chongqing, China; Department of Infectious Diseases, ChangdeXiangya Hospital, Central South University, Hunan, China; Population Research Unit, Faculty of Social Sciences, University of Helsinki, Helsinki, Finland; The Max Planck Institute for Demographic Research, Rostock, Germany; Department of Public Health Sciences, Stockholm University, Stockholm, Sweden

## Abstract

**Background:**

Stroke incidence has continued to increase recently in most countries. The roles of individual-level income on the incidence of overall stroke and its subtypes are still unknown, especially in low- and middle-income countries and the cross-national evidence is also limited. We explored the association between individual-level income and stroke incidence in Finland and China.

**Methods:**

Changde Social Health Insurance Database (*N*=571 843) and Finnish population register (*N*=4 046 205) data were used to calculate standard stroke incidence rates, which were employed to assess the absolute incidence difference between income quintiles. Cox regression was used to compare income differences in first-ever stroke incidence.

**Results:**

The highest income quintile had lower overall and subtype stroke incidence when compared to lower-income quintiles. The relative difference was more evident in hemorrhagic stroke incidence. After adjusting for age and employment status, the disparity of stroke incidence between the lowest and highest income quintiles was high among both men and women and in Finland and China. The disparity was particularly notable among men: in Finland, the hazard ratio (HR) for hemorrhagic stroke was 0.633 [95% confidence interval (95% CI) 0.576–0.696] and HR 0.572 (95% CI 0.540–0.606) for ischemic stroke. The respective figures were HR 0.452 (95% CI 0.276–0.739) and HR 0.633 (95% CI 0.406–0.708) for China.

**Conclusions:**

Individual-level income is related to overall and subtype stroke incidence. Future studies should explore the causal relationship between individual-level income and stroke incidence.

## Introduction

A stroke, which can be categorized as an ischemic or hemorrhagic stroke, is a neurological deficit that leads to an acute focal injury of the central nervous system because of a vascular cause.^1^ Stroke has become a leading cause of hospitalization and mortality in high-income as well as low- and middle-income countries.^2^ For example, in 2016, the age-standardized incidence of stroke was 353.70 cases per 100 000 persons in China, while the mortality for stroke was 132.84 cases per 100 000 persons.^3^ In Finland, the incidence and mortality from stroke were 61.7 and 41.6 per 100 000 persons in the same year, respectively.^4^ Furthermore, a stroke poses a significant burden for the organization and financing of health and social care because of the frequently severe cognitive and physical functioning consequences for individuals who survive.^2^ Because more than 90% of the stroke burden is arguably caused by modifiable risk factors,^5^ advancing our understanding of these risk factors for implementing effective interventions is necessary.

Lower income is also recognized as a risk factor for stroke. Research on the association between income and stroke has mainly focused on household-level income and demonstrated strong links with stroke prevalence,^6^ stroke care^7^ and stroke mortality.^8^ However, less is known about the association between individual income and stroke outcomes. While household income indicates general financial wellbeing, individual income also reflects the individual’s work status.^9^ As a result, these two indicators of income may have different effects on health. For instance, a previous study showed a stronger association between stroke mortality and individual income than between household income and stroke mortality.^10^

Even fewer studies have explored individual income and different subtypes of stroke.^11^^,^^12^ This is an important concern due to the differences in the etiologic mechanisms behind subtypes of stroke: ischemic stroke appears with regional loss of cerebral blood flow caused by stenotic or occluded cerebral vasculature while subarachnoid hemorrhage is caused by rupture of cerebral vasculature. The existing evidence on the associations between individual income and hemorrhagic stroke incidence is inconsistent. Some researchers reported that both ischemic and hemorrhagic stroke incidences were associated with individual income,^13^^,^^14^ while it has also been suggested that hemorrhagic stroke incidence is not associated with individual income.^11^^,^^12^

Additionally, the association between income and stroke incidence may vary between countries because of differences in intermediary factors, such as preventive measures and knowledge of potential risk factors. Therefore, lack of cross-national comparisons is an important gap in the literature. In particular, comparative studies including China are warranted, as previous studies have focused on high-income countries, and no study assessing the relevance of individual income on stroke incidence in China exists.

This study investigated the cross-country differences in the association between individual income and subtypes of stroke incidence in Finland and China utilizing health insurance and administrative register data.

## Methods

### Data sources

#### China

The Chinese data came from the Changde Health Insurance Database, which is a part of the Chinese National Health Insurance Database established in 2008 and managed by the Chinese National Health Commission. Changde is a prefecture in the northwest of the Hunan province in central China. The Database is an open cohort: individuals of all ages join this database whenever they register for a Chinese social health insurance scheme, including the Urban Resident Basic Medical Insurance (URBMI), Urban Employee Basic Medical Insurance (UEBMI) and the New Rural Cooperative Medical Scheme (NRCMS).^15^ The database contains basic information, such as age, sex, socioeconomic and employment status and hospital record information on admission diagnosis, discharge diagnosis and dates of entry and exit. In 2018, around 95% of citizens were covered by these insurance programs.^16^ Those who do not participate in these schemes include people who lack a hukou (household registration information); this group mainly consists of those who live in remote mountain areas.

As income levels are likely to be lower among young adults at the beginning of their working lives or still in education, we excluded individuals under the age of 30 from this study.

Because of migration and significant increase in coverage, the open database grew rapidly in 2012–19. In 2012, the database included 152 722 individuals over the age of 30 in Changde City and towns under Changde jurisdiction (Supplementary table S1). On 31 December 2019, the database comprised of 1 093 115 individuals (514 827 males and 578 288 females). In other words, 739 979 new individuals older than 30 were registered in this database over this period. Individuals were allowed to enter the analytical sample at any point after meeting the age criteria. Records for monthly income before taxes and working conditions were available for 571 843 participants (76.9% of all individuals above the age of 30 in the database). Individuals with missing data were excluded. This list-wise deletion caused no bias, as missingness was at random. Participants were censored at death or when they discontinue health insurance (because of the rules of the Chinese health insurance system, very few individuals discontinue their health insurance, and the reason for discontinuation is most often migration).

#### Finland

The Finnish full population dataset combined three annually updated data sources using the personal identification code that all residents receive: the Statistics Finland Labor Market Data file, the tax register and the hospital discharge registers maintained by the Finnish Institute for Health and Welfare. Of those 4 074 573 individuals above the age of 30 in 2012–19, the analyses were limited to those 4 046 205 with data on income, employment status and previous hospitalizations (see identification of stroke, below; 99.1% of all person-years). As in the Chinese sample, individuals entered the dataset at any stage of the follow-up upon when they met the minimum age requirement of 30 years. The participants were censored at permanent migration or death.

### Identification of stroke

Stroke patients were identified based on the International Classification of Disease 10th Reversion (ICD-10) from the hospital records in both countries. We used ICD codes I61–I62 to indicate hemorrhagic stroke and I63–I64 for ischemic stroke,^17^ while stroke without symptoms (8B21) was not included. Only first-incidence strokes were considered. In Changde, the first-incidence patients were identified based on a specific label for diagnostic information in the health insurance records. In Finland, as register data do not contain direct information on whether a stroke event is the first incidence, persons who had suffered a stroke within the 5 years preceding the study period were considered to have a history of stroke and were excluded from the analytical sample. This is a feasible solution and stroke is also relatively rare at younger ages.^18^

### Individual income

The local taxation bureau collects all citizens’ taxes and income information in China and sends it to other linked databases, such as the health insurance database. Income was classified relative to the individuals’ average pre-tax monthly income in given years reported by the Changde taxation bureau. All analyses were limited to participants with income information for all the years present in the data. By doing this, 22.1% of participants present in the data were excluded because of missing income information during the follow-up period.

In Finland, income was defined as the total annual taxable individual income (before taxes) in euros, measured on 31 December of each calendar year, derived from the Statistics Finland Labor Market Data file. Observations with no tax information were excluded (slightly under 1.0% of all observations). These individuals either had missing data or no taxable income during that calendar year.

Sex-specific annual income quintiles were then calculated for both Finland and China from these data.

### Employment status

In China, employment status was defined based on health insurance types and jobs. Employment status was measured annually at the end of each year. There are three most common types of insurance in China: NRCMS, UEBMI and URBMI. As only those employees currently or in the past could register for UEBMI, those registered in UEBMI were identified as employed or retired according to health insurance types and jobs labels. Participants with unemployed labels in job information were classified as unemployed, and those with other labels, such as veterans or students, were defined as others. We excluded 3% of individuals in the insurance registers with missing health insurance type.

In Finland, employment status was measured annually at the end of each calendar year. To match with the Chinese categorization, The Labor Market Data file information on employment status was grouped into employed, unemployed, retired, or other (e.g. student, at home, taking care of a family member and unknown).

### Data analysis

The standardized incidence of stroke was calculated by different stroke types, individual-level income level and sex using the standard 2019 world population from World Health Organization as the reference population.^19^

Cox proportional hazards (PHs) regression models were performed to estimate the association between individual-level income and stroke incidence. The 8-year follow-up began on 1 January 2012, or on 1 January of the year when the individual first entered the sample. Survival was defined as the time from baseline to the date of being first diagnosed with a stroke or the last time of follow-up. The crude model (Model 1) estimated hazard ratios (HRs) by income quintile for both sexes. Model 2 was further adjusted for age (continuous) and Model 3 for age and employment status (dummy variables). The PH assumption was tested using the Schoenfeld residuals test (Supplementary table S2). Some of the models did not meet the PH assumptions; these models were therefore additionally stratified by age. We also did a sensitivity analysis limited to the individual’s age between 30 and 60 years old to avoid the influence of lower salary after retirement on stroke. All analyses were conducted using Stata 16.0 and presented with 95% confidence intervals (95% CIs).

## Results

Out of the 317 263 Chinese men, 1174 were stroke patients, and out of 254 580 women, 890 were stroke patients (4.71 per 1000 person-years). In Finland, 57 325 individuals experienced a first-incidence stroke during the study period—27 910 men and 29 415 women (2.01 per 1000 person-years).

### Stroke incidence by income quintile

Tables 1 and 2 present the incidence rates of all strokes and subtypes of stroke over the 8-year follow-up for Chinese and Finnish residents by income quintile. Compared with men, women had lower overall and subtype of stroke incidence in most income quintiles in both China (e.g. hemorrhagic stroke incidence in lowest income quintile: male 1.82, 95% CI 0.68–2.96; female 1.26, 95% CI 0.98–1.54) and Finland (corresponding estimates 0.8, 95% CI 0.77–0.84 and 0.74, 95% CI 0.71–0.78). In both countries, stroke incidence in both sexes generally had an inverse relationship with income levels: higher income was associated with lower overall and subtype of stroke incidence.

**Table 1:** Eight-year subtype stroke incidence per 1000 person-years among males by income quintile

	China _(__*n*__of strokes=1174)_	Finland _(__*n*__of strokes=27 910)_
Income level	Incidence rate (95% CI)	Incidence rate (%) (95% CI)
Hemorrhagic stroke	0.67 (0.57–0.77)	0.50 (0.49–0.51)
1 (low)	1.82 (0.68–2.96)	0.80 (0.77–0.84)
2	1.70 (0.88–2.52)	0.78 (0.75–0.82)
3	0.49 (0.07–0.91)	0.41 (0.39–0.44)
4	0.43 (0.06–0.8)	0.25 (0.23–0.27)
5 (high)	0.26 (0.07–0.45)	0.24 (0.22–0.26)
Ischemic stroke	5.27 (4.96–5.58)	1.54 (1.52–1.56)
1 (low)	5.65 (3.95–7.35)	2.55 (2.49–2.61)
2	3.83 (1.83–5.81)	2.62 (2.56–2.68)
3	2.45 (1.02–3.88)	1.19 (1.15–1.23)
4	2.37 (1.42–3.32)	0.72 (0.69–0.75)
5 (high)	1.99 (0.64–3.34)	0.58 (0.56–0.61)
Any stroke	5.84 (5.52–6.17)	2.04 (2.02–2.06)
1 (low)	8.47 (5.37–11.57)	3.36 (3.30–3.43)
2	5.53 (1.83–9.23)	3.41 (3.35–3.48)
3	2.94 (1.32–4.56)	1.61 (1.56–1.66)
4	2.54 (1.70–3.38)	0.97 (0.93–1.01)
5 (high)	2.25 (0.64–3.86)	0.82 (0.79–0.86)

95% CI, 95% confidence interval.

**Table 2:** Eight-year subtype stroke incidence per 1000 person-years among females by income quintile

	China _(__*n*__of strokes=890)_	Finland _(__*n*__of strokes=5494)_
Income level	Incidence rate (%) (95% CI)	Incidence rate (%) (95% CI)
Hemorrhagic stroke	0.66 (0.34–0.98)	0.49 (0.48–0.50)
1 (low)	1.26 (0.98–1.54)	0.74 (0.71–0.78)
2	0.60 (0.08–1.12)	0.81 (0.78–0.84)
3	0.50 (0.06–0.94)	0.47 (0.44–0.49)
4	0.64 (0.03–1.25)	0.23 (0.21–0.25)
5 (high)	0.33 (0.02–3.86)	0.18 (0.17–0.20)
Ischemic stroke	3.96 (3.65–4.27)	1.49 (1.47–1.51)
1 (low)	5.80 (3.82–7.78)	2.41 (2.35–2.46)
2	2.38 (0.89–3.87)	2.87 (2.80–2.93)
3	2.26 (0.10–4.42)	1.27 (1.23–1.31)
4	2.19 (0.82–3.56)	0.55 (0.52–0.57)
5 (high)	0.72 (0.64–0.80)	0.36 (0.34–0.38)
Any stroke	4.17 (3.86–4.48)	1.99 (1.97–2.01)
1 (low)	7.06 (3.82–10.30)	3.16 (3.10–3.23)
2	3.98 (1.24–6.72)	3.70 (3.63–3.77)
3	2.98 (0.10–5.86)	1.74 (1.69–1.79)
4	2.83 (0.82–4.84)	0.78 (0.75–0.81)
5 (high)	0.42 (0.24–0.60)	0.54 (0.52–0.57)

95% CI, 95% confidence interval.

In China, there were relatively larger income gaps in incidence between middle and middle-high (Levels 3 and 4) income categories for women than men. Among women in Finland, those in the second lowest income group fared worse than those in the lowest group. In both countries, the difference between the lowest and highest income groups was more considerable in ischemic stroke incidence than hemorrhagic stroke incidence (e.g. in China, hemorrhagic stroke: men 7.0-fold and women 3.87-fold; ischemic stroke: men 2.83-fold and women 1.58-fold).

### Cox regression results

The income gradient was clear among men in China and Finland and for both overall and subtype of stroke. For example, prior to adjusting for age and employment status in China (table 3, Model 1), the overall stroke incidence among men in the lowest income group was 525% higher than that in the highest quintile (HR 0.16, 95% CI 0.13–0.20) (table 3). Among Finnish men, the HR of overall stroke incidence in the highest income group was 0.245 compared to those in the lowest income group (95% CI 0.234–0.257). Among Chinese men, the income gradient was weaker in hemorrhagic stroke incidence than in ischemic stroke (table 3), while among Finnish men the difference between the two stroke subtypes was slightly less prominent. In both China and Finland, after adjusting for age and employment status explained roughly half of the association between income and stroke among men and women (tables 3 and 4, Model 3).

**Table 3:** HRs for the incidence of stroke among men by income quintile from 2012 to 2019

	China			Finland			
	Model 1^a^	Model 2^b^	Model 3^c^	Model 1^a^	Model 2^b^	Model 3^c^	
Income level	HR (95% CI)	HR (95% CI)	HR (95% CI)	HR (95% CI)	HR (95% CI)	HR (95% CI)	
Hemorrhagic stroke							
1 (low)	1.0	1.0	1.0	1.0	1.0	1.0	
2	0.445 (0.316–0.626)	0.580 (0.410–0.822)	0.596 (0.419–0.847)	0.985 (0.929–1.046)	0.853 (0.803–0.905)	0.872 (0.822–0.926)	
3	0.442 (0.336–0.580)	0.554 (0.419–0.731)	0.574 (0.432–0.763)	0.517 (0.481–0.555)	0.714 (0.663–0.768)	0.783 (0.728–0.842)	
4	0.210 (0.151–0.290)	0.315 (0.224–0.441)	0.331 (0.234–0.468)	0.311 (0.286–0.339)	0.560 (0.512–0.612)	0.657 (0.600–0.720)	
5 (high)	0.207 (0.137–0.310)	0.405 (0.253–0.647)	0.452 (0.276–0.739)	0.297 (0.272–0.324)	0.526 (0.480–0.576)	0.633 (0.576–0.696)	
Ischemic stroke							
1 (low)	1.0	1.0	1.0	1.0	1.0	1.0	
2	0.738 (0.637–0.855)	1.153 (0.995–1.338)	1.162 (1.001–1.348)	1.038 (1.004–1.072)	0.881 (0.853–0.911)	0.880 (0.851–0.910)	
3	0.568 (0.498–0.647)	0.797 (0.698–0.910)	0.797 (0.698–0.911)	0.469 (0.450–0.489)	0.721 (0.691–0.752)	0.754 (0.722–0.786)	
4	0.335 (0.290–0.386)	0.660 (0.570–0.764)	0.673 (0.581–0.780)	0.283 (0.269–0.297)	0.621 (0.590–0.654)	0.686 (0.651–0.723)	
5 (high)	0.150 (0.117–0.191)	0.536 (0.406–0.708)	0.633 (0.481–0.834)	0.230 (0.218–0.243)	0.506 (0.479–0.536)	0.572 (0.540–0.606)	
Any stroke							
1 (low)	1.0	1.0	1.0	1.0	1.0	1.0	
2	0.676 (0.590–0.775)	1.028 (0.896–1.181)	1.053 (0.916–1.210)	1.024 (0.995–1.054)	0.873 (0.848–0.899)	0.877 (0.852–0.903)	
3	0.547 (0.485–0.616)	0.755 (0.669–0.853)	0.786 (0.696–0.889)	0.481 (0.464–0.498)	0.719 (0.693–0.746)	0.760 (0.732–0.788)	
4	0.307 (0.269–0.351)	0.582 (0.508–0.666)	0.618 (0.538–0.709)	0.289 (0.277–0.302)	0.604 (0.578–0.632)	0.676 (0.646–0.708)	
5 (high)	0.161 (0.130–0.200)	0.520 (0.408–0.663)	0.607 (0.473–0.778)	0.245 (0.234–0.257)	0.511 (0.487–0.536)	0.585 (0.557–0.615)	

HR, hazard ratio; 95% CI, 95% confidence interval.

a Model 1: crude model.

b Model 2: adjusting for age.

c Model 3: adjusting for age and employment status.

**Table 4:** HRs for the incidence of stroke among women by income quintile from 2012 to 2019

	China			Finland		
	Model 1^a^	Model 2^b^	Model 3^c^	Model 1^a^	Model 2^b^	Model 3^c^
Income level	**HR** (**95% CI)**	**HR** (**95% CI)**	**HR** (**95% CI)**	**HR** (**95% CI)**	**HR** (**95% CI)**	**HR** (**95% CI)**
Hemorrhagic stroke						
1 (low)	1.0	1.0	1.0	1.0	1.0	1.0
2	0.555 (0.324–0.952)	0.650 (0.394–1.157)	0.728 (0.424–1.251)	1.194 (1.157–1.232)	1.057 (1.024–1.090)	1.062 (1.029–1.096)
3	0.306 (0.156–0.596)	0.498 (0.253–0.978)	0.722 (0.357–1.459)	0.528 (0.507–0.549)	0.911 (0.875–0.948)	0.943 (0.907–0.982)
4	0.300 (0.485–0.485)	0.461 (0.284–0.749)	0.549 (0.336–0.899)	0.225 (0.213–0.238)	0.797 (0.753–0.842)	0.881 (0.832–0.932)
5 (high)	0.090 (0.033–0.246)	0.209 (0.075–0.581)	0.405 (0.133–1.232)	0.149 (0.139–0.159)	0.649 (0.607–0.695)	0.743 (0.694–0.796)
Ischemic stroke						
1 (low)	1.0	1.0	1.0	1.0	1.0	1.0
2	0.538 (0.441–0.656)	0.653 (0.535–0.796)	0.786 (0.643–0.961)	1.174 (1.142–1.207)	1.046 (1.018–1.076)	1.052 (1.023–1.082)
3	0.249 (0.190–0.327)	0.399 (0.303–0.524)	0.814 (0.613–1.080)	0.55 (0.532–0.569)	0.924 (0.893–0.956)	0.954 (0.922–0.987)
4	0.320 (0.269–0.380)	0.497 (0.418–0.592)	0.644 (0.539–0.768)	0.244 (0.233–0.256)	0.794 (0.758–0.833)	0.865 (0.824–0.908)
5 (high)	0.061 (0.039–0.095)	0.146 (0.093–0.229)	0.365 (0.225–0.509)	0.171 (0.162–0.181)	0.672 (0.636–0.711)	0.751 (0.709–0.795)
Any stroke						
1 (low)	1.0	1.0	1	1.0	1.0	1.0
2	0.541 (0.448–0.653)	0.779 (0.644–0.942)	0.786 (0.649–0.951)	1.095 (1.034–1.160)	0.996 (0.940–1.055)	1.005 (0.948–1.065)
3	0.255 (0.198–0.328)	0.801 (0.614–1.046)	0.825 (0.632–1.076)	0.625 (0.585–0.669)	0.952 (0.890–1.020)	0.986 (0.920–1.057)
4	0.320 (0.272–0.377)	0.621 (0.526–0.734)	0.639 (0.540–0.756)	0.306 (0.281–0.333)	0.760 (0.694–0.833)	0.822 (0.748–0.903)
5 (high)	0.066 (0.044–0.099)	0.360 (0.232–0.559)	0.388 (0.250–0.604)	0.246 (0.224–0.270)	0.692 (0.627–0.764)	0.760 (0.685–0.844)

HR, hazard ratio; 95% CI, 95% confidence interval.

a Model 1: crude model.

b Model 2: adjusting for age.

c Model 3: adjusting for age and employment status.

Among Chinese women, income had a notable impact on overall and subtype of stroke before adjusting for age and employment status, similar to the results among Chinese men (table 4, Model 1). As among men, differences between the income groups attenuated in both countries after controlling for age and employment status (table 3, Model 3). In the fully adjusted Model 3, the point estimates for any, ischemic and stroke hemorrhagic stroke still implied a smaller risk among those in the higher income groups. However, the difference between the lowest income references and the higher income groups was not consistently statistically significant.

## Discussion

This large population-based study found that individual income was associated with overall and subtype stroke incidence in Finland and China and among both men and women. The relationship was not driven by age or employment status. Given the notable economic and welfare losses caused by stroke, an effective prevention strategy is necessary, particularly for China.^20^ To our knowledge, this study is the first to investigate the relationship between individual income and stroke incidence in China and compared it with a high-income country.

The main findings are consistent with the previous studies related to individual-level income and stroke incidence. A large study from Germany (*N* = 42 966) showed that, compared with a lower-income group, individuals with higher individual-level income have lower overall stroke incidence before adjusting for employment status, and the effect of income on stroke incidence is more evident in males [males: HR 0.75, 95% CI (0.73–0.77); females: HR 0.96, 95% CI (0.93–0.99)].^21^ The results concerning ischemic stroke incidence are also similar to those in prior studies.^13^ However, contrary to our results, which show that income related to hemorrhagic stroke incidence, one study found individual-level income measured in four quartiles irrelevant for hemorrhagic stroke incidence.^12^

Various behavioral and psychosocial mechanisms may explain the association between income and stroke. First, individuals with lower income have a higher prevalence of cardiometabolic risk factors, such as smoking prevalence,^22^ which are risk factors for stroke incidence.^23^^,^^24^ Lower income is also related to lower control in the workplace and less social support, which are crucial social risk factors for cardiovascular disease.^25^ Additionally, lower income is associated with psychiatric conditions, such as depression and anxiety.^23^^,^^26^^,^^27^ These psychiatric conditions may then contribute to cardiovascular disease incidence through biomedical routes, such as sedentariness, inflammation and metabolic syndrome^28^^,^^29^ or mediators like psychosocial stress.^30^

This study demonstrated that stroke incidence is higher in Chinese than in Finnish residents, which is also in accordance with previous estimates.^31^ Several factors may explain the overall difference between the countries. The incidence of stroke is reported to be highly correlated with national per capita income, probably because of healthier life habits like less smoking behavior and universal access to healthcare in the high-income country.^20^ Prior studies support this notion, as the prevalence of several risk factors for strokes, such as diabetes and hypertension, have increased notably in China over the last 15 years, especially among lower-income individuals.^32^

Moreover, the timing of the establishment of stroke intervention-related policies may play a role in the overall difference in stroke incidence between China and Finland. In China, the Ministry of Health China Stroke Prevention Project Committee was founded in 2011 to prevent stroke.^33^ Meanwhile, in Finland, intervention projects to control cardiovascular mortality, including stroke, through lifestyle changes and drug treatment have been in place since 1972.^34^ Other factors, such as inadequate health services and insufficient health literacy in low- and middle-income countries, may also explain the stroke incidence disparity.^35^ Low- and medium-income countries are encouraged to establish national-level stroke intervention policies to confront the increased trend of stroke incidence, alleviating poverty and also having a long-term benefit in controlling stroke incidence.

Except for the association between stroke and income, our study also indicated that there may be a stronger association between stroke incidence and income in China than in Finland. This may relate to the differences in the size of the gap between the richest and the poorest (e.g. the Gini index) in both countries. According to World Bank data, in 2016, the Gini index was 38.5 in China and 27.1 in Finland.^36^^,^^37^ In other words, the gap between the income levels is steeper in China than in Finland, which is a potential explanation for our findings.

### Strengths and limitations

The strength of this study is the sizeable, longitudinal data. Using administrative register data from Finland and China enabled us to reduce biases related to non-response, loss to follow-up and self-reporting. Also, as we noted above, it is the first study comparing the relationship between individual income and stroke incidence in China.

There are still some limitations. First, the missing data in the Chinese databases is a concern. Around 23% (22.1% missed income and 0.7% employment information) of the participants in the Chinese databased are missing income and employment status information. This study used deletion to deal with missing data, participants with missing data were excluded. However, this approach has two main disadvantages: discarding valuable information may lead to loss of power and may potentially cause bias.^38^ However, the latter is not a major concern in the case of this study, as the missingness was considered to be random. The results are also consistent with the previous evidence on average family income and stroke incidence in China.^39^ Moreover, this database only includes people who received treatment, but people in the lower-income group may have lower health service utilization for stroke. Therefore, the results may have underestimated the relationship between lower income and stroke incidence.

The proxy method of a 5-year wash-out period used in Finland means that individuals with a stroke that occurred more than 5 years ago could be included in this study. Considering individuals with a history of stroke have a higher risk of subsequent events, this proxy method is therefore likely to narrow the gap between Finland and China.^40^

Income is strongly correlated with age: young adults and students are likely to have low incomes, and income is likely to again drop after retirement. This study was limited to those above the age of 30 and we adjusted for the role of age. Additionally, we tested whether the results were driven by older adults with low income by limiting the analyses to ages 30–60 (Supplementary table S5). We observed a similar income gradient in stroke incidence among working age individuals also.

It is unclear whether the results derived from the Changde Social Health Insurance can be generalized to all China. However, the incidence of stroke in Changde is very similar to that observed in China as a whole^40^ and change is also similar has socioeconomic and sex distributions to the other 687 cities in China—most of which are county-level cities (a kind of city category in China). Thus, we believe the results from Changde, a county-level city, to be generalizable to China as a whole.

To ensure commensurability, only age and employment status are controlled in this study. Other explanatory confounders, such as education levels, are not adjusted for. We performed Cox regression in the data analysis, which means that some unmeasured confounders were not controlled. The results only implicate an association but no causal relationship. Further studies are needed to explore the causal relationship between income and stroke incidence.

## Conclusion

In this study, we found that individual-level income was associated with overall and subtype of stroke incidence, and this association was stronger in China. The relationship between individual-level income and stroke incidence was more substantial in males than females. The evidence from this study implicates that alleviating poverty-related policy could play a role in reducing stroke incidence, which is helpful for further policymaking. More research is needed to explore the causal relationship between income and stroke incidence.

## Supplementary Material

ckad035_Supplementary_Data

## Data statement

Data cannot be shared for ethical/privacy reasons. The data underlying this article cannot be shared publicly due to it is dataset from government, which need permissions before sharing.
