# Supplementary material for: The relationship of income on stroke incidence in Finland and China
Source: Eur J Public Health. 2023 Apr 22;33(3):360–5. doi: 10.1093/eurpub/ckad035 (PMC10234641; doi:10.1093/eurpub/ckad035)
Supplement: ckad035_Supplementary_Data [file ckad035_supplementary_data.docx]

**Supplementary Table 1. Demographic information of participants in China and Finland**

|  |  |  | China | | Finland | |
| --- | --- | --- | --- | --- | --- | --- |
|  |  |  | Men | Women | Men | Women |
| 2012  **(Baseline)** | Individuals |  | 67,916 | 49,563 | 1,092,044 | 1,076,871 |
|  | Age (mean [SD]) |  | 45.33 (14.72) | 41.18 (14.29) | 45.15 (9.01) | 45.42 (9.02) |
|  | Income (mean [SD]) * |  | 4158.69 (2812.72) | 3667.80 (2296.73) | 2520.71 (1506.51) | 2143.1 (1058.56) |
|  | Employment status (N [%]) | Employed | 53,792 (79.20) | 37,885 (76.44) | 6,762,931 (78.00) | 6,759,826 (79.42) |
|  |  | unemployed | 76 (0.11) | 134 (0.27) | 937,444 (10.81) | 719584 (8.45) |
|  |  | Retired | 14,033 (20.66) | 11,541 (23.29) | 562,991 (6.49) | 445,548 (5.23) |
|  |  | Others | 15 (0.00) | 3 (0.00) | 407,478 (4.70) | 586,213 (6.89) |
| 2013 | |  | 153,973 | 123,849 | 1,090,305 | 1,073,852 |
| 2014 | |  | 168,809 | 135,324 | 1,087,730 | 1,070,949 |
| 2015 | |  | 187,535 | 146,892 | 1,085,850 | 1,068,307 |
| 2016 | |  | 261,376 | 210,746 | 1,083,183 | 1,063,139 |
| 2017 | |  | 294,579 | 237,894 | 1,080,206 | 1,057,737 |
| 2018 | |  | 308,556 | 248,572 | 1,076,991 | 1,052,144 |
| 2019 | |  | 317,263 | 254,580 | 1,074,535 | 1,048,172 |

*Monthly income measured in CNY in China and in EUR in Finland (derived from annual income).

***p*-value > 0.05

**Supplementary Table 2 Schoenfeld Residuals test results**

|  | **Men** | | | **Women** | | |
| --- | --- | --- | --- | --- | --- | --- |
| **Income Level** | **Model 1^a^** | **Model 2^b^** | **Model 3^c^** | **Model 1^a^** | **Model 2^b^** | **Model 3^c^** |
| **China** |  |  |  |  |  |  |
| Hemorrhagic stroke | 0.410 | 0.181* | 0.226* | 0.818 | 0.875 | 0.223 |
| Ischemic stroke | 0.884 | 0.236 | 0.108* | 0.259 | 0.051 | 0.226* |
| Both kinds of stroke | 0.381 | 0.123* | 0.081* | 0.208 | 0.427* | 0.292* |
| **Finland** |  |  |  |  |  |  |
| Hemorrhagic stroke | 0.903 | 0.815 | 0.760 | 0.865 | 0.941 | 0.391 |
| Ischemic stroke | 0.548 | 0.066 | 0.071 | 0.996 | 0.905 | 0.788 |
| Both kinds of stroke | 0.512 | 0.067 | 0.191* | 0.993 | 0.955 | 0.703 |

a Model 1: income

b Model 1 + age

c Model 1+age+ employment status

*Age stratified

**Supplementary Table 3. Hazard ratios for the incidence of stroke among men by income quintile from 2012 to 2019 (limited to 30-60 years old)**

|  | **China** | | | | | | **Finland** | | | | | |
| --- | --- | --- | --- | --- | --- | --- | --- | --- | --- | --- | --- | --- |
|  | **Model 1^a^** | | **Model 2^b^** | | **Model 3^c^** | | **Model 1^a^** | | **Model 2^b^** | | **Model 3^c^** | |
| **Income Level** | **HR** | **95% CI** | **HR** | **95% CI** | **HR** | **95% CI** | **HR** | **95% CI** | **HR** | **95% CI** | **HR** | **95% CI** |
| Hemorrhagic stroke |  |  |  |  |  |  |  |  |  |  |  |  |
| 1 (low) | 1.0 |  | 1.0 |  | 1.0 |  | 1.0 |  | 1.0 |  | 1.0 |  |
| 2 | 1.000 | (0.642, 1.559) | 1.057 | (0.678, 1.648) | 1.076 | (0.688, 1.682) | 0.616 | (0.556,0.682) | 0.643 | (0.581,0.712) | 0.746 | (0.671,0.829) |
| 3 | 0.513 | (0.299, 0.879) | 0.791 | (0.460, 1.361) | 0.815 | (0.472, 1.407) | 0.476 | (0.428,0.531) | 0.530 | (0.476,0.590) | 0.714 | (0.631,0.808) |
| 4 | 0.820 | (0.514, 1.310) | 0.909 | (0.569, 1.453) | 0.940 | (0.581, 1.522) | 0.361 | (0.322,0.405) | 0.407 | (0.363,0.457) | 0.565 | (0.495,0.646) |
| 5 (high) | 0.590 | (0.352, 0.987) | 0.450 | (0.269, 0.754) | 0.465 | (0.273, 0.791) | 0.359 | (0.321,0.402) | 0.371 | (0.331,0.415) | 0.522 | (0.457,0.597) |
| Ischemic stroke |  |  |  |  |  |  |  |  |  |  |  |  |
| 1 (low) | 1 |  | 1 |  | 1 |  | 1.0 |  | 1.0 |  | 1.0 |  |
| 2 | 0.864 | (0.764, 0.977) | 0.929 | (0.821, 1.051) | 1.088 | (0.960, 1.233) | 0.738 | (0.689,0.790) | 0.779 | (0.728,0.834) | 0.856 | (0.797,0.919) |
| 3 | 0.238 | (0.197, 0.288) | 0.393 | (0.324, 0.476) | 0.468 | (0.386, 0.569) | 0.520 | (0.483,0.560) | 0.598 | (0.556,0.644) | 0.740 | (0.681,0.804) |
| 4 | 0.317 | (0.267, 0.376) | 0.365 | (0.307, 0.433) | 0.536 | (0.448, 0.641) | 0.429 | (0.397,0.463) | 0.504 | (0.467,0.545) | 0.637 | (0.583,0.695) |
| 5 (high) | 0.422 | (0.361, 0.492) | 0.313 | (0.268, 0.365) | 0.501 | (0.425, 0.591) | 0.395 | (0.366,0.427) | 0.419 | (0.388,0.453) | 0.534 | (0.489,0.585) |
| Both kinds of stroke |  |  |  |  |  |  |  |  |  |  |  |  |
| 1 (low) | 1 |  | 1 |  | 1 |  | 1.0 |  | 1.0 |  | 1.0 |  |
| 2 | 0.873 | (0.775, 0.983) | 0.937 | (0.832, 1.056) | 1.086 | (0.963, 1.225) | 0.696 | (0.657,0.736) | 0.732 | (0.692,0.775) | 0.824 | (0.777,0.874) |
| 3 | 0.256 | (0.214, 0.307) | 0.420 | (0.351, 0.504) | 0.496 | (0.413, 0.595) | 0.504 | (0.475,0.536) | 0.574 | (0.540,0.610) | 0.731 | (0.682,0.784) |
| 4 | 0.351 | (0.299, 0.411) | 0.402 | (0.343, 0.471) | 0.573 | (0.485, 0.677) | 0.404 | (0.380,0.431) | 0.470 | (0.441,0.500) | 0.610 | (0.567,0.657) |
| 5 (high) | 0.433 | (0.373, 0.502) | 0.321 | (0.278, 0.373) | 0.496 | (0.424, 0.581) | 0.382 | (0.359,0.407) | 0.402 | (0.377,0.428) | 0.524 | (0.487,0.565) |

HR, hazard ratio; CI, confidence interval.

1. **Model 1:** crude model
2. **Model 2:** adjusting for age
3. **Model 3:** adjusting for age and employment status

**Supplementary Table 4. Hazard ratios for the incidence of stroke among women by income quintile from 2012 to 2019 (limited to 30-60 years old)**

|  | **China** | | | | | | **Finland** | | | | | |
| --- | --- | --- | --- | --- | --- | --- | --- | --- | --- | --- | --- | --- |
|  | **Model 1^a^** | | **Model 2^b^** | | **Model 3^c^** | | **Model 1^a^** | | **Model 2^b^** | | **Model 3^c^** | |
| **Income Level** | **HR** | **95% CI** | **HR** | **95% CI** | **HR** | **95% CI** | **HR** | **95% CI** | **HR** | **95% CI** | **HR** | **95% CI** |
| Hemorrhagic stroke |  |  |  |  |  |  |  |  |  |  |  |  |
| 1 (low) | 1.0 |  | 1.0 |  | 1.0 |  | 1.0 |  | 1.0 |  | 1.0 |  |
| 2 | 1.381 | (0.788, 2.422) | 1.572 | (0.894, 2.766) | 1.409 | (0.804, 2.472) | 0.903 | (0.801,1.017) | 0.915 | (0.812,1.031) | 1.039 | (0.912,1.184) |
| 3 | 0.333 | (0.142, 0.784) | 0.489 | (0.205, 1.167) | 0.418 | (0.176, 0.993) | 0.619 | (0.544,0.703) | 0.631 | (0.555,0.718) | 0.780 | (0.670,0.909) |
| 4 | 0.095 | (0.022, 0.406) | 0.177 | (0.041, 0.772) | 0.135 | (0.031, 0.586) | 0.493 | (0.431,0.564) | 0.528 | (0.462,0.605) | 0.658 | (0.561,0.772) |
| 5 (high) | 0.001 | - | 0.001 | - | 0.000 | - | 0.519 | (0.455,0.591) | 0.539 | (0.473,0.614) | 0.674 | (0.576,0.789) |
| Ischemic stroke |  |  |  |  |  |  |  |  |  |  |  |  |
| 1 (low) | 1.0 |  | 1.0 |  | 1.0 |  | 1.0 |  | 1.0 |  | 1.0 |  |
| 2 | 1.193 | (0.996, 1.428) | 1.590 | (1.327, 1.906) | 1.520 | (1.266, 1.825) | 0.749 | (0.681,0.824) | 0.763 | (0.694,0.840) | 0.955 | (0.860,1.060) |
| 3 | 0.500 | (0.397, 0.629) | 1.035 | (0.820, 1.305) | 1.015 | (0.805, 1.281) | 0.544 | (0.491,0.602) | 0.562 | (0.508,0.622) | 0.825 | (0.729,0.934) |
| 4 | 0.463 | (0.366, 0.586) | 1.474 | (1.160, 1.873) | 1.456 | (1.146, 1.840) | 0.432 | (0.388,0.481) | 0.476 | (0.428,0.530) | 0.707 | (0.621,0.806) |
| 5 (high) | 0.371 | (0.288, 0.479) | 1.001 | (0.768, 1.304) | 1.185 | (0.894, 1.571) | 0.367 | (0.328,0.410) | 0.391 | (0.350,0.437) | 0.584 | (0.511,0.668) |
| Both kinds of stroke |  |  |  |  |  |  |  |  |  |  |  |  |
| 1 (low) | 1.0 |  | 1.0 |  | 1 |  | 1.0 |  | 1.0 |  | 1.0 |  |
| 2 | 1.209 | (1.019, 1.436) | 1.593 | (1.341, 1.893) | 1.527 | (1.283, 1.817) | 0.803 | (0.745,0.865) | 0.816 | (0.757,0.879) | 0.983 | (0.906,1.067) |
| 3 | 0.485 | (0.389, 0.606) | 0.982 | (0.785, 1.228) | 0.966 | (0.772, 1.208) | 0.570 | (0.527,0.617) | 0.586 | (0.541,0.635) | 0.804 | (0.730,0.885) |
| 4 | 0.431 | (0.342, 0.543) | 1.329 | (1.051, 1.682) | 1.315 | (1.039, 1.663) | 0.453 | (0.416,0.492) | 0.493 | (0.453,0.536) | 0.683 | (0.617,0.756) |
| 5 (high) | 0.339 | (0.263, 0.436) | 0.867 | (0.668, 1.126) | 1.015 | (0.772, 1.338) | 0.421 | (0.387,0.458) | 0.444 | (0.408,0.483) | 0.618 | (0.558,0.684) |

HR, hazard ratio; CI, confidence interval.

1. **Model 1:** crude model
2. **Model 2:** adjusting for age
3. **Model 3:** adjusting for age and employment status
